# A highly sensitive, selective and renewable carbon paste electrode based on a unique acyclic diamide ionophore for the potentiometric determination of lead ions in polluted water samples

**DOI:** 10.1039/d0ra01435d

**Published:** 2020-05-06

**Authors:** M. A. Zayed, Walaa H. Mahmoud, Ashraf A. Abbas, Aya E. Ali, Gehad G. Mohamed

**Affiliations:** Chemistry Department, Faculty of Science, Cairo University 12613 Giza Egypt ggenidymohamed@sci.cu.edu.eg; Egypt Nanotechnology Center, Cairo University El-Sheikh Zayed, 6^th^October 12588 Giza Egypt

## Abstract

Due to the toxicity of lead(ii) to all living organisms as it destroys the central nervous system leading to circulatory system and brain disorders, the development of effective and selective lead(ii) ionophores for its detection is very important. In this work, 1,3-bis[2-(*N*-morpholino)acetamidophenoxy]propane (BMAPP), belonging to acyclic diamides, was applied as a highly selective lead(ii) ionophore in a carbon paste ion selective electrode for the accurate and precise determination of Pb(ii) ions even in the presence of other interfering ions. Factors affecting the electrode's response behavior were studied and optimized. Scanning electron microscopy (SEM), energy dispersive X-ray (EDX) and FT-IR spectroscopy were used for studying the morphology and response mechanism of the prepared sensor. The lipophilicity of the used ionophore, which contributes to the mechanical stability of the sensor, was studied using the contact angle measurement technique. The selectivity coefficients obtained by the separate solution method (SSM) and fixed interference method (FIM) confirmed the selectivity of the proposed sensor for Pb(ii) ions. The proposed sensor exhibited a Nernstian slope of 29.96 ± 0.34 mV per decade over a wide linear range of 5 × 10^−8^ to 1 × 10^−1^ mol L^−1^ and detection limit of 3 × 10^−8^ mol L^−1^ for 2 months with a fast response time (<10 s) and working pH range (2.5–5.5). To further ensure the practical applicability of the sensor, it was successfully applied for the lead(ii) ion determination in different water samples and the obtained data showed an agreement with those obtained by atomic absorption spectroscopy. In addition, it was successfully applied for the potentiometric titration of Pb(ii) against K_2_CrO_4_ and Na_2_SO_4_.

## Abstract 2

Due to the toxicity of lead(ii) to all living organisms destroying the central nervous system and leading to circulatory system and brain disorders, the development of effective and selective lead(ii) ionophores for its detection is very important.

## Graphical abstract

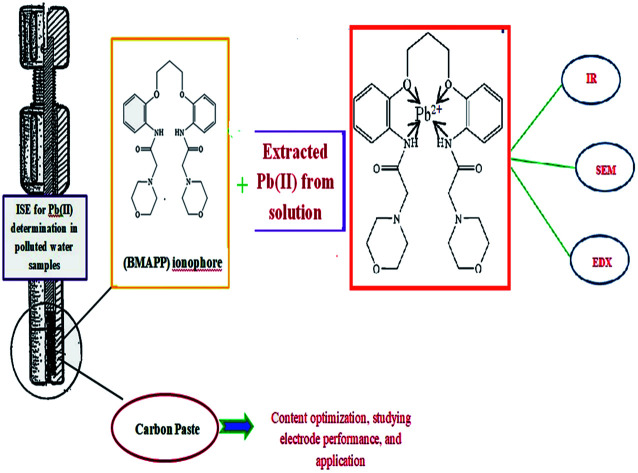

## 1. Introduction

Among all the heavy metals, lead(ii) is the most common and abundant pollutant in the environment. The permissible limit for Pb is 0.05 mg L^−1^ and if its concentration surpasses the permissible limit in water, the water becomes toxic for human usage.^1–3^ It is hazardous to all living creatures because it is disposed to build up in the bones if it is absorbed at a rate above 300 μg per day. It has serious influences on the cardiovascular, immune, central nervous and reproductive systems. Additionally, it influences the kidney and inhibits the maturity of the nervous system, which results in possibly permanent learning and behavior disorders in children.^1,4^ Moreover, lead concentrations as minute as 10 ppb can reduce intelligence and neurological advancements and lead to many health disorders such as nephropathies, gastrointestinal tract alterations, reproductive dysfunction, and hemotoxic effects upon the long term exposure.^5^ Lead was extensively in use more than any other metal^6^ but due to its severe toxicity, lead was drained away from some products such as pigments, electrical and electronic products, gasoline, and solders.^7^ However, its use in lead-acid batteries (LABs) despite of other innovative battery technologies was due to their maturity, cost effectiveness, safety, and applicability.^8^ On the other hand, LABs and the rapid growth of lead-related industries, such as lead smelting, recycling, and wire rope were the main cause of lead pollution in China resulting in common public health problems putting children's health in danger.^9,10^ Bearing in mind the toxic properties of lead and the rigorous regulations of its distribution, it becomes crucial to detect and determine lead in trace amounts in the environment, especially in water used for consumption and production. Moreover, the need for consistent and cost-effective analyses of lead(ii) in aqueous media and organisms becomes more serious to certify environmental safety.^3,11^ The reported techniques for the determination of Pb(ii) such as spectrophotometry,^12,13^ differential pulse polarography,^14^ spectrofluorimetry,^15^ and ion chromatography^16^ have disadvantages of high cost, which restricts their application for routine analysis. The main challenge in the ISE field is to improve the sensitivity and selectivity by searching for a suitable modifier.

Ion-selective electrodes (ISEs) are commonly used in clinical, industrial and environmental analysis.^17^ The relatively low cost and low maintenance make this modest design of chemical sensors advantageous over other analytical techniques. More importantly, measurements with ISEs are done in the potentiometric mode leaving the measured sample intact in means of chemical composition; thus further analysis of the same sample by other methods is possible.^17^ ISEs are suitable for reliable monitoring of pollutants in natural waters if the requirements of high selectivity and low detection limit are fulfilled. Taking into consideration the toxic properties of lead and rigorous regulations of its distribution, ISEs become a valuable tool for the determination of this component.^17^ Despite the immense effort towards obtaining ISEs devoted to the determination of the ionized lead at low concentrations in environmental samples, there is no industrially available ISE for the determination of trace concentrations of Pb^2+^.^17^

Ion selective electrodes (ISEs) are considered important for the determination of different pollutants in the environment in a selective manner.^18,19^ Carbon paste electrode (CPE) is considered as a heterogeneous carbon electrode of composite nature made of graphite powder as an electrical conductor embedded in a suitable binder that provides the mechanical stability to the paste.^20^ CPEs are characterized by low background current, ease of fabrication and their surface renewability.^5^ The ISE technique offers many advantages such as low cost, non-destructive analysis, portability, and quick and simple operation without any need for sample pre-treatment,^4,21,22^ In this research, a new carbon paste ion selective electrode modified with 1,3-bis[2-(*N*-morpholino)acetamidophenoxy]propane as an ionophore was fabricated for the selective and sensitive determination of lead ions and the electrochemical response was studied in buffered solutions of lead (pH = 4.5, acetate buffer). Selectivity and parameters affecting the electrode response such as ionophore content, plasticizer type, pH, temperature and response time were evaluated and optimized. The proposed sensor was utilized for the determination of Pb(ii) in different water samples in the presence of other ions.

## 2. Experimental

### 2.1. Materials and reagents

Analytical grade reagents were used in this study. Solutions were prepared from a stock solution of 0.1 mol L^−1^ Pb(ii), prepared from a sufficient quantity of lead nitrate supplied from Prolabo in bidistilled water and buffered at pH = 4.5 using acetate buffer. The working solutions were prepared daily by suitable dilution of the stock solution. All other solutions used in interference studies were prepared from analytical grade chloride salts purchased from El Nasr company. *o*-Nitrophenyloctyl ether (*o*-NPOE) was supplied by Fluka, while dioctyl phthalate (DOP) and dibutyl phthalate (DBP) were supplied by BDH. 2-Fluorophenyl-2-nitrophenyl ether (FFNE), tricresyl phosphate (TCP) and graphite powder (synthetic 1–2 μm) were supplied by Sigma Aldrich. K_2_CrO_4_ and Na_2_SO_4_ used in the potentiometric titration were supplied by Adwick.

### 2.2. Real water samples

Different real water samples were collected. They included formation water (Amry deep (7) (sample 1) and Falak (11) (sample 2), from Western Desert, Agiba Petroleum Company, Egypt), underground tap water supplied from Manshat El-Kanater network (sample 3) and river water (sample 4; the intake of Nekla station).

### 2.3. Apparatus

The potential measurements were carried out using a digital Hanna pH mV meter (model 8417). Silver–silver chloride double-junction reference electrode (HANNA, HI 5311) in conjugation with the prepared sensor under study was used. Jenway 3505 pH meter was used for pH measurements. Digital burette was used for the potentiometric titration of Pb(ii). Automatic pipettes (Socorex Swiss (50–200 μL and 200–1000 μL)) were used to measure the very small volumes whereas glass micropipettes were used to measure the large volumes. For surface analysis, SEM Model Quanta 250 FEG (Field Emission Gun) attached with an EDX Unit (Energy Dispersive X-ray Analyses) with accelerating voltage 30 KV, magnification 14× up to 1 000 000 and resolution for Gun.1n, The Egyptian Mineral Resources Authority Central Laboratories Sector, was used. The FT-IR spectra were measured on a PerkinElmer 1650 spectrometer (4000–400 cm^−1^) using the potassium bromide pellet technique at the Microanalytical Center, Cairo University, Egypt. Contact angle analyzer of model T200 manufacture by Biolin Scientific under conditions of sessile drop recipe, droplet distilled water volume 4 μm and measure time 10 s was used.

### 2.4. Procedure

#### 2.4.1. Preparation of the ionophore

**Scheme 1:**
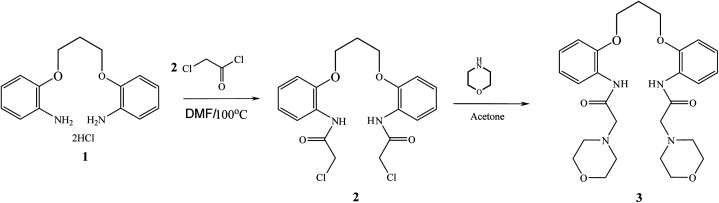
Preparation of 1,3-bis[2-(*N*-morpholino)acetamidophenoxy]propane ionophore (3).

The ionophore 1,3-bis[2-(*N*-morpholino)acetamidophenoxy]propane (3) (Scheme 1) was prepared in two steps as reported.^23^ Firstly, 1,3-bis[(2-chloroacetamido)phenoxy]propane (2) was prepared by the reaction of 1,3-bis(2-aminophenoxy)propane dihydrochloride (1) with chloroacetyl chloride in DMF at 100 °C and then, a mixture of 1,3-bis(2-aminophenoxy)propane (2) and excess morpholine with few drops of triethylamine in acetone was heated under reflux for 1 h. The solvent was then removed *in vacuo*. The obtained solid, which is the desired ionophore, was washed with cold water and crystallized from ethanol as colorless crystals.

#### 2.4.2. Preparation of modified carbon paste electrodes (MCPEs)

250 mg pure graphite powder and 5–20 mg of the prepared ionophore were transferred to a mortar and mixed well with a plasticizer (0.1 mL of *o*-NPOE, TCP, DOP, DBP or FFNE). The modified paste was filled in a Teflon holder serving as the electrode body with a stainless steel rod inserted through the center of the holder for electrical contact and was kept in distilled water for 24 h before use. To obtain fresh surface, the stainless-steel screw was pushed frontward and a new carbon-paste surface was polished on a filter paper to get a glossy novel surface.^24^

#### 2.4.3. Potential measurements

In order to calibrate the newly prepared MSPEs, the prepared sensor was immersed in conjunction with a reference electrode in a 25 mL beaker encompassing 10 mL aliquot of Pb(ii) solution (pH = 4.5, acetate buffer) having concentrations ranging from 5 × 10^−8^ to 1 × 10^−1^ mol L^−1^ with continuous stirring and the potential was recorded after steadying to ±1 mV. Then, a calibration graph was made by plotting the recorded potentials as a function of −log[Pb(ii)]. The resulting graph was used for the subsequent determination of unknown lead concentration.^25,26^

#### 2.4.4. Selectivity coefficient determination

In this work, selectivity coefficients of the electrode toward different cationic species (M^*n*+^) were evaluated by the separate solution method (SSM), (0.001 mol L^−1^ solutions of Pb(ii) and interfering ions) by comparing the potential of two solutions; the selectivity coefficient was determined using the following equation:^27^1
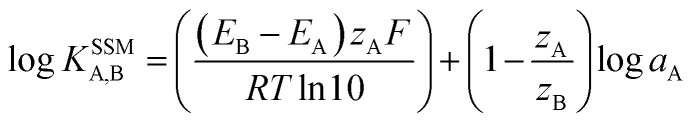
where *E*_A_ and *E*_B_ are the measured potential of Pb(ii) and interfering ions, respectively; *z*_A_ and *z*_B_ are the charge numbers of the primary ion, A, and of the interfering ion, B; and *a*_A_ is the activity of the primary ion, A. In eqn (1), it is considered that *a*_A_ = *a*_B_ and *E*_A_ and *E*_B_ are the responses of the electrode to primary and interfering ions, respectively.

In addition, the selectivity coefficients of the interfering species were evaluated by the fixed interference method (FIM). In this manner, the MCPE and the reference electrode were placed in a 50.0 mL beaker containing 0.001 mol L^−1^ interference ion solution and the Pb(ii) ion concentration was varied over a wide range (from 1.0 × 10^−9^ to 1.0 × 10^−1^ mol L^−1^) while the interfering ion (M^*n*+^) concentration was kept constant. The solution was stirred magnetically and the cell potential was recorded. The pH of the total test solutions was constant around 4.5. The emf values obtained were plotted against the logarithm of the activity of the primary ion and the selectivity coefficient was determined using the following equation:^28^2
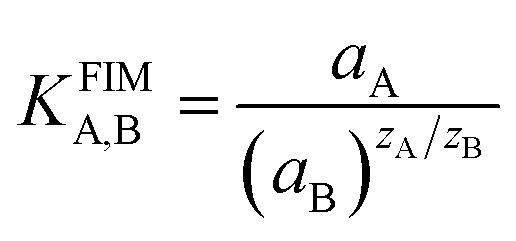


In eqn (2), *a*_A_ and *a*_B_ are the activities of the primary ion and the interfering ion, respectively.

#### 2.4.5. Surface analysis

The energy dispersive X-ray analyzer (EDX) and scanning electron microscope (SEM) were used for the surface analysis of the paste at 4000× magnifications for the proposed sensor before and after interaction with lead ions.

## 3. Results and discussion

### 3.1. Optimization of the carbon paste electrode components

The ionophore based ISEs can be used to quantify more than 70 different analytes including inorganic and organic ions, and even some nonionic species like phenol derivatives and nonionic surfactants.^29^ This “omnivorosity” of the ionophore based ISEs is due to the large variety of the ionophores: neutral or charged lipophilic agents capable of selective binding with the respective analytes. The ion to ionophore interactions can be selective for several different reasons: the size of the analyte ion may be a perfect fit for the cavity in the ionophore structure or the functional polar groups of the ionophore may specifically bind to the analyte ion, *etc.* This selectivity in complexation translates into the selectivity of the potentiometric response of the electrode. The selectivity, sensitivity and response time of an ion-selective sensor is mainly related to the stability of the formed complex between the ion and ionophore.^30^ As evident from Scheme 1, the present ionophore has a crown ether like cavity that results in the formation of a stable chelate with two 5-membered rings and one 6-membered ring between the used ionophore and lead ion resulting in a large formation constant and fast exchange kinetics that was confirmed by SEM, EDX and IR studies as will be discussed in further sections.

The amount of ionophore in the paste conformation and the properties of the pasting liquid have major influence on the selectivity, sensitivity, and linear range of the selective carbon paste electrode.^31^ In case the amount of the used ionophore in the paste is adequate, a rational chemical equilibrium occurs at the electrode/solution border that is responsible for the electrode potential. However, if such material is in excess, the ratio of ionic sites to the ionophore will change, leading to substandard functioning.^32^ Different electrodes containing different amounts (5–20 mg) of BMAPP ionophore were prepared and calibrated with the Pb(ii) solution and the slope was revealed. It was obvious that the optimum ionophore content was found to be 10 mg with respect to the slope, linear range and regression as indicated in Table 1.

**Table 1:** Effect of the composition of carbon paste ingredients on the electrode performance

Electrode no.	Composition of various components in carbon pastes (amount in mg)			Electrode characteristics		
	BMAPP ionophore, mg	Plasticizer (100 mg)	Graphite, mg	Slope ± SD, mV per decade	Linear range, mol L^−1^	Regression
1	5	TCP	250	31.98 ± 0.73	5 × 10^−7^ to 1 × 10^−1^	0.9990
**2**	**10**	**TCP**	**250**	**29.96 ± 0.34**	**5 × 10^−8^ to 1 × 10^−1^**	**0.9996**
3	15	TCP	250	28.53 ± 0.84	1 × 10^−6^ to 1 × 10^−1^	0.9988
4	20	TCP	250	27.92 ± 0.50	5 × 10^−6^ to 1 × 10^−1^	0.9989
5	10	*o*-NPOE	250	31.16 ± 1.51	5 × 10^−7^ to 1 × 10^−1^	0.9982
6	10	DBP	250	31.05 ± 0.62	5 × 10^−6^ to 1 × 10^−1^	0.9983
7	10	DOP	250	30.80 ± 1.01	1 × 10^−5^ to 1 × 10^−1^	0.9991
8	10	FFNE	250	24.50 ± 0.81	1 × 10^−5^ to 1 × 10^−1^	0.9990

The kind of pasting liquid (plasticizer) has a great effect on determining the carbon paste electrode characteristics because its nature affects the dielectric constant of the carbon paste and the mobility of the ions. In addition, it enables homogenous solubilization and modification of the distribution constant of the used ionophore.^30,33^ In the investigation for a suitable plasticizer, five plasticizers, namely, TCP, *o*-NPOE, DOP, DBP and FFNE, were used in sample electrodes to figure out the plasticizer with the best response. It was found that TCP as a solvent mediator produced the best response with respect to the slope, linear range, repeatability and regression as shown in Table 1. Electrode no. 2 has been chosen for further study.

### 3.2. Selectivity studies

**Fig. 1:**
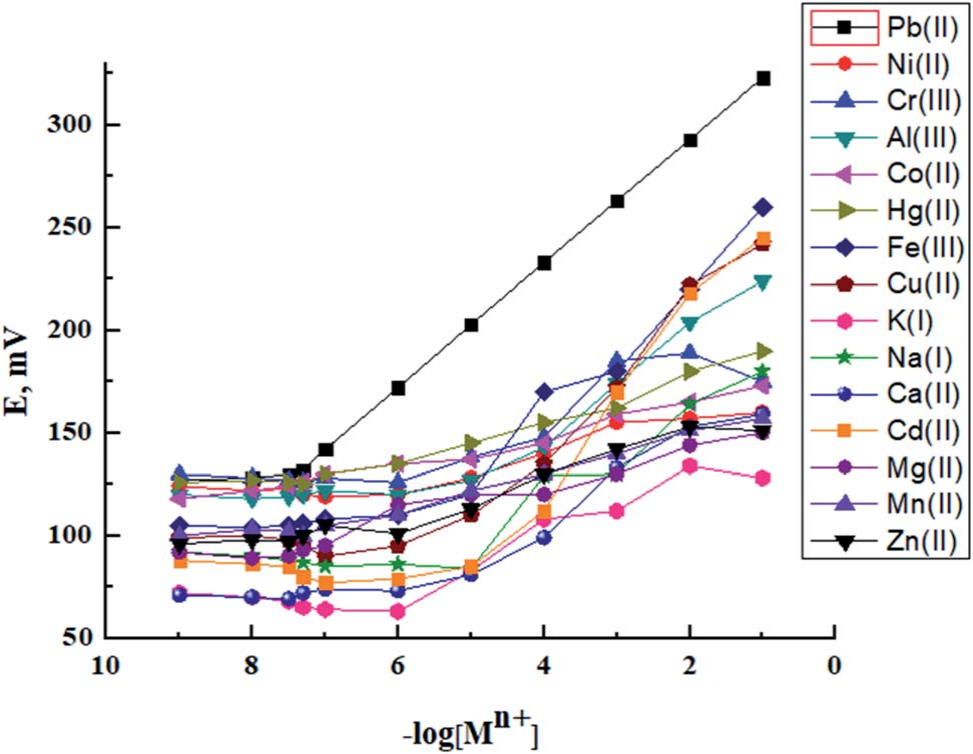
Potential response of the proposed sensor for different metal ions.

The ionophore has the major control on the selectivity of an ISE membrane. The affinity between the analyte and the ionophore and the ion-split between two immiscible phases establish the base mechanism of the potentiometric ion sensors.^34^ In preliminary experiments, BMAPP ionophore was applied for the preparation of carbon paste electrodes of the same composition as electrode no. 2, given in Table 1, for a variety of metal ions and their potential responses are shown in Fig. 1. It was established that among all the investigated cations, Pb(ii) ion showed the Nernstian potential response over a wide concentration range and this can be assigned to the selective behavior and more interaction of the ionophore with Pb(ii) over other metal ions as well as the fast exchange kinetics of the resulting complex.^35,36^ In addition, the dissimilarities in the ionic size and the permeability of the tested ions hinder their interference.^37^

In this work, selectivity coefficients of the proposed electrode towards different cationic species (M^*n*+^) were evaluated by using both of the separate solution method (SSM), (0.001 mol L^−1^ of Pb(ii) and interfering ions)^31,38^ and the fixed interference method (FIM) where selectivity coefficients were evaluated graphically from potential measurements in solutions containing 0.001 mol L^−1^ of the interfering ions and varying concentrations of Pb(ii) ions.^32,39^ The measured selectivity coefficients are given in Table 2. It can be seen that the selectivity coefficient values are much smaller than 1.0, which indicate a good discrimination of the proposed sensor for Pb(ii) ions from the other metal ions.

**Table 2:** Selectivity coefficients of the Pb(ii) sensor in presence of other ions

Foreign ion	*K* ^SSM^_Pb(ii),B_	*K* ^FIM^_Pb(ii),B_
Ni^2+^	3.13 × 10^−4^	3.16 × 10^−4^
Cd^2+^	9.91 × 10^−4^	4.83 × 10^−3^
Co^2+^	4.25 × 10^−4^	9.25 × 10^−4^
Cu^2+^	9.90 × 10^−4^	2.24 × 10^−4^
Mn^2+^	9.88 × 10^−5^	8.53 × 10^−5^
Zn^2+^	1.15 × 10^−4^	9.95 × 10^−5^
Cr^3+^	3.14 × 10^−4^	3.01 × 10^−5^
Al^3+^	1.35 × 10^−4^	1.60 × 10^−4^
Fe^3+^	2.14 × 10^−4^	1.58 × 10^−4^
Hg^2+^	7.94 × 10^−4^	9.65 × 10^−4^
Ca^2+^	5.77 × 10^−5^	5.01 × 10^−4^
Mg^2+^	4.58 × 10^−5^	8.03 × 10^−5^
Na^+^	4.60 × 10^−2^	8.01 × 10^−2^
K^+^	1.14 × 10^−2^	7.89 × 10^−2^

### 3.3. SEM, EDX and IR analyses

The performance of a modified carbon paste electrode depends on the selective extraction of the target ion with the aid of the plasticizer. The response mechanism may be attributed to the complex formation at the sensor surface by the extraction of Pb(ii) ions from the solution into the paste of a suitable modifier content and plasticizer during the measurement.^40^ This stability was also confirmed by SEM, EDX and IR studies. In an attempt to relate the potentiometric response to surface morphology, energy dispersive X-ray analysis (EDX) and scanning electron microscopy (SEM), which is considered as an important technique to illustrate the surface morphology of sensors,^41–44^ were used.

**Fig. 2:**
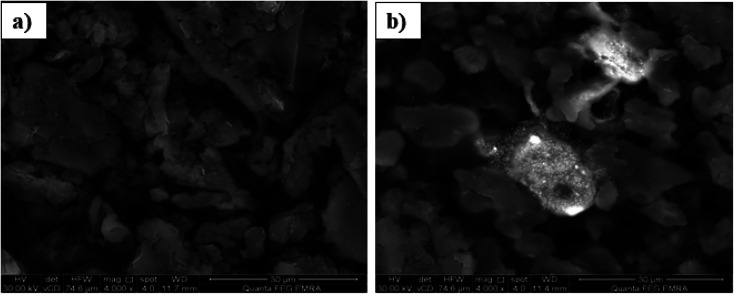
SEM image of the surface of the proposed sensor (250 mg graphite, 100 mg TCP and 10 mg BMAPP ionophore) (a) before and (b) after soaking in 1.0 × 10 ^−3^ mol L^−1^ Pb(ii) ion for 1 h at 25 °C.

**Fig. 3:**
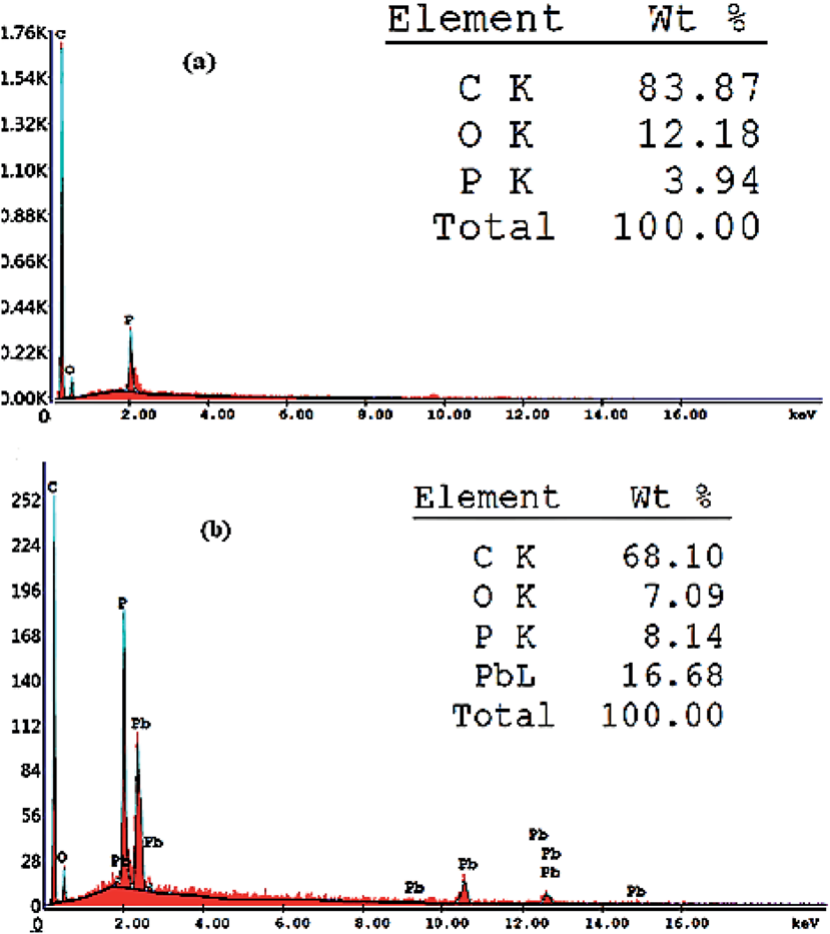
EDX analysis showing weight% of different elements present in the proposed sensor (a) before and (b) after soaking in 1.0 × 10^−3^ mol L^−1^ Pb(ii) ion solution for 1 h at 25 °C.

The proposed sensor was prepared according to its optimum composition and then soaked in 10^−3^ mol L^−1^ of lead ion solution for 1 hour. As shown in Fig. 2, the sensor surface is homogeneous and permeable and includes grains that facilitate the Pb(ii) ion diffusion, which is proved by the development of illuminated spots filling the cavities between these carbon grains and alteration of surface morphology after soaking and can be explained by the complex formation between the Pb(ii) ions and the used modifier. This mechanism was also supported by EDX analysis, which gave quantitative information about the surface composition as shown in Fig. 3. IR spectra confirmed these data. It was revealed that the absorption (NH) band at 3271 cm^−1^ was shifted to 3250 cm^−1^ and the etheric oxygen band was shifted to a lower frequency from 1165 cm^−1^ to 1149 cm^−1^ while the other ionophore band positions remained unchanged, which in turn confirmed the complex formation between Pb(ii) ion and the used ionophore through coordination *via* the NH and etheric oxygens.

### 3.4. Effect of pH

**Fig. 4:**
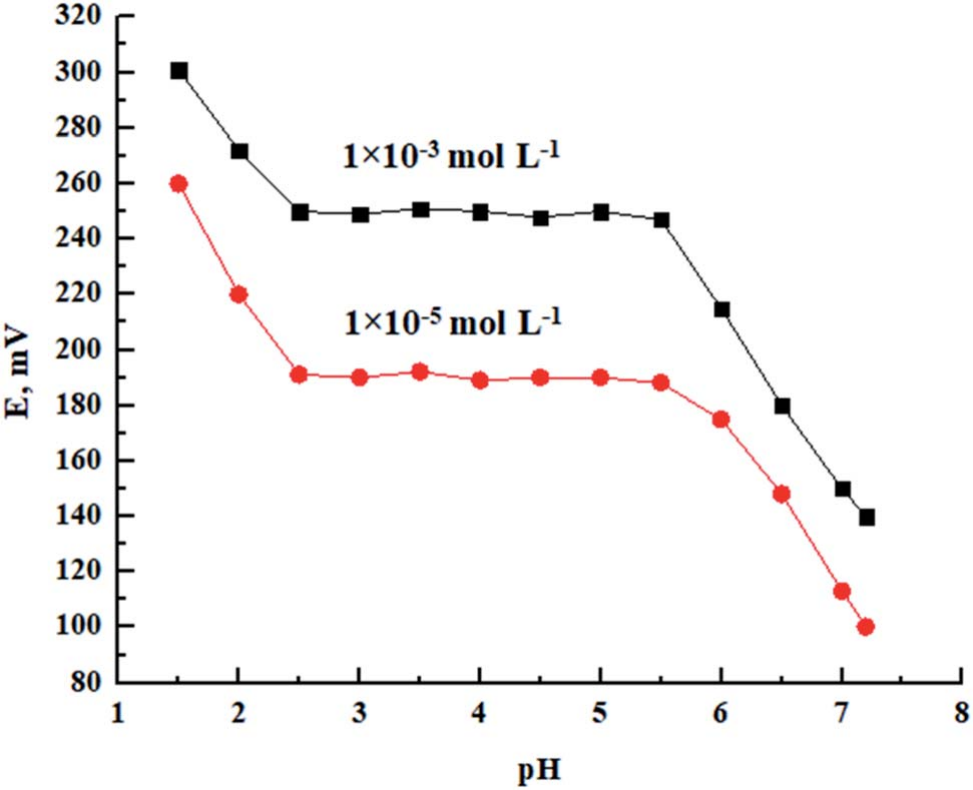
Effect of pH at two different Pb(ii) concentrations on the potentiometric response of the proposed Pb(ii) sensor.

As illustrated in Fig. 4, the response of the proposed sensor at 1.0 × 10^−5^ and 1 × 10^−3^ mol L^−1^ of Pb(ii) ion at different pH values was studied. Dilute NaOH/HNO_3_ solutions were used to adjust the solution pH. The electrode response was pH independent in the range of 2.5–5.5. At a pH higher than 5.5, the hydrolysis of lead ions into lead hydroxide (*K*_sp_ = 1.42 × 10^−20^) took place, which decreased the potentiometric response considerably. On the other hand, at a pH lower than 2.5, H^+^ ions can replace the Pb(ii) ions and cause interference by ligand protonation at such high concentration of hydrogen ions.^18,45^

### 3.5. Response time and reversibility

**Fig. 5:**
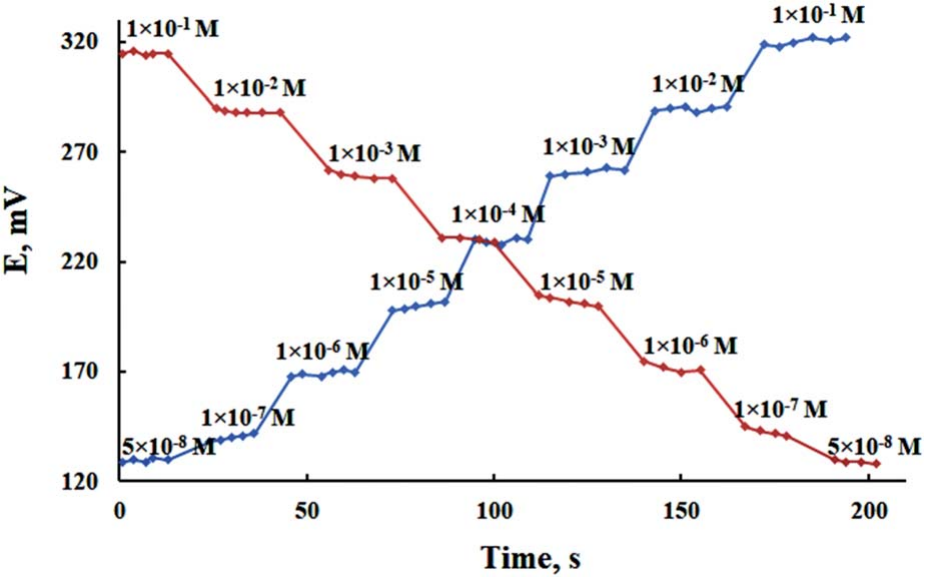
Dynamic response of the proposed MCPE for step changes of Pb(ii) concentration from low-to-high and high-to-low.

Dynamic response time, which is defined as the average time required for a sensor to reach potential within ±1 mV of the final equilibrium value, is an important factor for any sensor. The critical emf response of the electrode was assessed according to IUPAC recommendations.^46^ As shown in Fig. 5, it is evident that the response time of the proposed sensor was less than 10 s for Pb(ii) concentrations in the range of 5.0 × 10^−8^ to 1.0 × 10^−1^ mol L^−1^ with response stability up to 5 min. In order to study the reversibility of the used MCPE, the potential was recorded in the sequence of high-to-low concentrations of Pb(ii) ion. The results shown in Fig. 5 revealed that the potential response was reversible and these observations implied that no memory effect on the electrode response was noticed.^47^

### 3.6. Effect of temperature

In order to study the influence of temperature on the EMF response of the proposed sensor, calibration curves were constructed at different temperatures covering the range 10–70 °C. As shown in Table 3, the slope of the calibration graph of the proposed sensor was Nernstian up to 60 °C of the test solution and the linear concentration range was almost unchanged, which means that the investigated electrode can be used up to 60 °C without noticeable deviation from the Nernstian behavior. However, temperatures higher than 60 °C caused a significant deviation from the theoretical values and this can be attributed to a damage of the electrode surface caused by some leaching in the paste matrix lowering the response.^48^ To calculate the isothermal coefficient (d*E*°/d*t*)_cell_ of the cell, the standard cell potentials, 
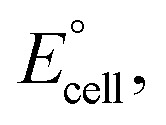
 were determined at different temperatures from the respective calibration graphs as the intercept of these plots at −log[Pb(ii), mol L^−1^] = 0 and were plotted *versus* (*t* – 25), where *t* was the temperature of the test solution in °C applying Antropov's equation.^24–26,33,40^ The isothermal coefficient value was 6.1 × 10^−5^ V °C^−1^, which is a small value revealing the high thermal stability of the studied sensor within the investigated temperature range.

**Table 3:** Effect of temperature on the potentiometric response of the proposed Pb(ii) sensor

*T* (°C)	Slope (mV per decade)	Linear range (mol L^−1^)	*R* ^2^
10	30.68	5 × 10^−8^ to 1 × 10^−1^	0.9991
20	30.43	5 × 10^−8^ to 1 × 10^−1^	0.9997
30	29.61	5 × 10^−8^ to 1 × 10^−1^	0.9996
40	29.54	5 × 10^−8^ to 1 × 10^−1^	0.9997
50	28.14	5 × 10^−8^ to 1 × 10^−1^	0.9992
60	27.04	5 × 10^−8^ to 1 × 10^−1^	0.9991
70	19.89	1 × 10^−6^ to 1 × 10^−1^	0.9912

### 3.7. Ionophore lipophilicity, lifetime, homogeneity, reproducibility and detection limit

**Fig. 6:**
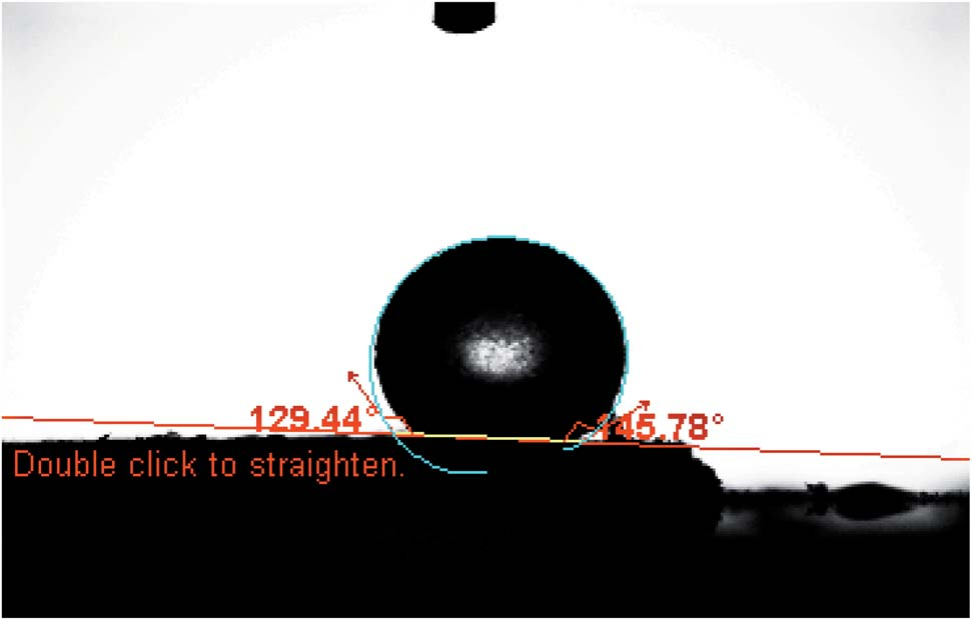
Contact angle formed by a sessile water drop on a smooth homogenous surface of the used ionophore.

Constant potentials and long lifetime depend mainly on the lipophilicity and good solubility of the used ionophore in the paste matrix, which ensures the absence of any ionophore leaching.^49^ As shown in Fig. 6, contact angle measurement was used to measure the ionophore lipophilicity and the average contact angle was 137.086°, which is much larger than 90° corresponding to low wettablility *i.e*., lipophilicity of the used ionophore.^50^ It was observed that the paste could be in use for a period of two months without significant deviation from the Nernstian behaviour. The surface of the MCPE was renewed by polishing it on a filter paper before calibration. It was also rinsed carefully in distilled water to remove the memory effects.^40^

To test the paste homogeneity, the proposed electrode was applied for Pb(ii) measurement in a 1.0 × 10^−3^ mol L^−1^ Pb(ii) solution. The measurement was repeated five times and after each measurement the electrode surface was renewed by squeezing a little carbon paste out of the holder and a fresh surface was smoothed on a piece of weighing paper. The average potential was 265 mV with a RSD% value of 0.95, which was reasonable.

On the other hand, to evaluate the reproducibility of the proposed sensor, a series of pastes (five) with the optimum composition, as shown in Table 1, was prepared and the responses of the prepared electrodes were tested for Pb(ii) ion concentration of 1.0 × 10^−3^ mol L^−1^. The results showed that the proposed electrodes have good reproducibility with a RSD% value of 1.98. The limit of detection, which was evaluated according to the IUPAC recommendations^46^ was found to be 3.0 × 10^−8^ mol L^−1^ for Pb(ii) ions.

### 3.8. Analytical application

The concentration of Pb(ii) was measured in real water samples including formation water, underground tap water, river water and sea water samples. The pH of each sample was adjusted by NaOH and HNO_3_ to the desired pH. An aliquot of standard solutions was added to the sample and the Pb(ii) concentrations were determined. Table 4 summarizes the results obtained for all the water samples and the recovery% for the applied electrode was satisfactory, in spite of the presence of other cations, because of the high selectivity and low detection limit of the constructed Pb(ii) sensor. A very good correspondence between the spiked and experimentally obtained results was observed.

**Table 4:** Determination of lead(ii) in spiked water samples and the comparison of the results with those obtained by ICP-AAS

Sample no.	Taken, mg mL^−1^	Found, mg mL^−1^		RSD%		Recovery%	
		Sensor calibration	ICP-AAS	Sensor calibration	ICP-AAS	Sensor calibration	ICP-AAS
1	0.0021	0.0021	0.0020	1.46	2.33	100.0	95.24
	0.0207	0.0205	0.0202	1.22	1.75	99.03	97.58
	0.2070	0.2087	0.2060	0.80	1.66	100.8	99.52
2	0.0021	0.0020	0.0020	2.01	2.25	95.24	95.24
	0.0207	0.0208	0.0202	1.20	1.53	100.5	97.58
	0.2070	0.2093	0.2051	0.98	0.41	101.1	99.08
3	0.0021	0.0020	0.0020	2.22	1.98	95.24	95.24
	0.0207	0.01997	0.0201	1.38	1.47	96.47	97.10
	0.2070	0.2010	0.2039	0.72	0.78	97.10	98.50
4	0.0021	0.0021	0.0021	1.97	1.84	100.0	100.0
	0.0207	0.0206	0.0207	2.02	1.95	99.52	100.0
	0.2070	0.2031	0.2017	0.95	0.78	98.12	97.44

**Fig. 7:**
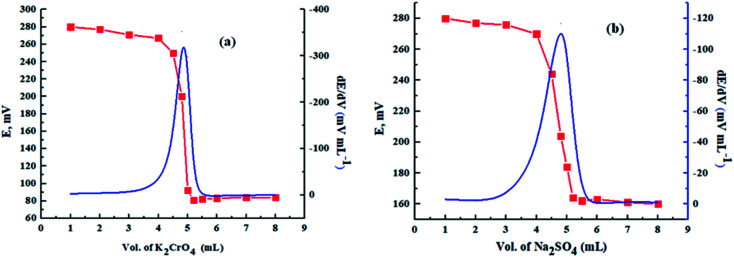
S-shape and first derivative titration curves for the potentiometric titration of 5.0 mL (1.0 × 10^−2^) mol L^−1^ Pb(NO_3_)_2_ solution against (a) 1.0 × 10^−2^ mol L^−1^ of K_2_CrO_4_ and (b) 1.0 × 10^−2^ mol L^−1^ of Na_2_SO_4_.

In addition, as shown in Fig. 7, the proposed sensor was used successfully as an indicator electrode in the potentiometric titration of 5 mL of 1.0 × 10^−2^ mol L^−1^ Pb(ii) solution, adjusted at pH = 4.5 using acetate buffer, against standard solutions of 1.0 × 10^−2^ mol L^−1^ K_2_CrO_4_ and Na_2_SO_4_.

### 3.9. Comparative study

The main performance characteristics (linear range, detection limit, lifetime, working pH, slope and selectivity) of some of the lead-selective electrodes from the literature^51–56^ against the data of the proposed lead ISEs are listed in Table 5. As can be seen, the proposed lead-selective electrode exhibited improved characteristics of response such as concentration range, detection limit, selectivity coefficients for potential interfering ions (especially for Hg(ii), which was considered as a main interferent in the previously reported Pb(ii) ISEs) and response time. However, they have nearly the same Nernstian slopes and working pH ranges. In addition, all of the listed electrodes are based on the PVC membrane, which suffers from increased system impedance and the electrode response time.^57^ This work is based on carbon paste potentiometric electrode which is simple, cheap and renewable. So, it is apparent that this electrode is superior to previously reported electrodes in most cases as it can be used in a wider concentration range with an enhanced sensitivity and selectivity for Pb(ii) ions from a wide variety of other heavy metal ions (which is more proper for industrial samples) with a very fast response time and a fairly long lifetime. It must be noted that there are voltammetric and fluorescent sensors^58,59^ that show enhanced selectivity and sensitivity in the determination of Pb(ii) ions, but they are to some extent expensive and complicated.

**Table 5:** Comparison between the proposed sensor and previously published sensors

Ref.	Modifier	Slope, mV per decade	Detection limit, mol L^−1^	Linear range, mol L^−1^	pH range	Response time, s	Life time	Interfering ions with 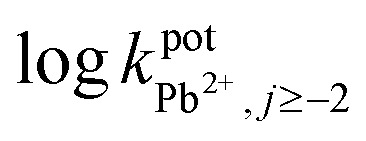
51	Poly(*m*-phenylenediamine) microparticles	29.8	6.31 × 10^−7^	3.16 × 10^−6^ to 3.16 × 10^−2^	3.0–5.0	14	5 months	Hg(ii), Ag(i), Na(i), K(i) and NH_4_(i)
52	I: 1,3-bis(*N*′-benzoylthioureido)benzene	I: 31.50	I: 1.6 × 10^−6^	I: 4.0 × 10^−6^ to 1.0 × 10^−2^	2.2–6.0	14	I: 10 weeks	Cu(ii) and Cd(ii)
	II: 1,3-bis(*N*′-furoylthioureido)benzene	II: 30.00	II: 1.9 × 10^−6^	II: 5.0 × 10^−6^ to 1.0 × 10^−2^			II: 14 weeks	Cu(ii)
53	Polyaminoanthraquinone (PAAQ) microparticles	28.90	7.76 × 10^−7^	2.5 × 10^−6^ to 1.0× 10^−1^	2.8–5.2	12	4 months	Hg(ii), Ag(i), Au(iii) and Cu(ii)
54	Butyl-3-(2-phenylhydrazono) indolin-2-one	29.50	3.2× 10^−7^	7.7 × 10^−7^ to 1.0 × 10^−1^	3.7–6.3	6	10 weeks	Hg(ii), Ag(i) and Fe(ii)
55	Quinolyl phenylhydrazone	28.70	6.0 × 10^−7^	1.0 × 10^−6^ to 1.0 × 10^−1^	3.0–6.0	14	2 months	Ag(i), Cu(ii), Hg(ii) and Fe(iii)
56	5,11,17,23-Tetra-*tert*-butyl-25,26,27,28-tetrakis-(diphenylphosphinoylmethoxy)calix[4]arene	28.00	1.4 × 10^−6^	1.0 × 10^−5^ to 1.0 × 10^−2^	3.5–5.0	17	56 days	Mn(ii), Zn(ii), Cd(ii), Ag(i), La(iii) and Ca(ii)
This study	1,3-Bis[2-(*N*-morpholino)acetamidophenoxy]propane	29.96	3.0 × 10^−8^	5.0 × 10^−8^ to 1.0 × 10^−1^	2.5–5.5	10	2 months	Na(i) and K(i)

## 4. Conclusion

A new Pb(ii) ion selective carbon paste electrode was developed by simple incorporation of 1,3-bis[2-(*N*-morpholino)acetamidophenoxy]propane as an ionophore. It was found that the proposed sensor showed an enhancement in the performance of the Pb(ii) ISE in comparison to the other previously reported electrodes. This simple and easily fabricated Pb(ii) sensor can serve as an indicator electrode in the potentiometric titration of Pb(ii) against K_2_CrO_4_ and Na_2_SO_4_ and can also be used to establish Pb(ii) ion concentration in real water samples containing various interfering metal ions in a selective and sensitive manner.

## Conflicts of interest

There are no conflicts to declare.

## Supplementary Material

## References

[cit1] Arfin T., Tarannum A. (2019). J. Membr. Sci..

[cit2] Needleman H. L., Bellinger D. (1991). Annu. Rev. Public Health.

[cit3] Huang M. R., Ding Y. B., Li X. G. (2013). Analyst.

[cit4] Hussain R., Khan M. Q., Khan A. A. (2019). Groundwater for Sustainable Development.

[cit5] Wang Y., Wu Y., Xie J., Hu X. (2013). Sens. Actuators, B.

[cit6] Heskel D. L. (1983). Curr. Anthropol..

[cit7] Mansson N., Bergback B., Sorme L. (2009). J. Ind. Ecol..

[cit8] Liu W., Tian J., Chen L., Guo Y. (2017). Environ. Pollut..

[cit9] Chen K., Huang L., Yan B., Li H., Sun H., Bi J. (2014). Environ. Sci. Technol..

[cit10] Lv Y., Sun T., Rang W. (2013). Chin. Prev. Med..

[cit11] Liu Y., Liu Y., Gao Y., Wang P. (2019). Sens. Actuators, B.

[cit12] Ebrahimzadeh H., Asgharinezhad A. A., Moazzen E., Amini M. M., Sadeghi O. (2015). J. Food Compos. Anal..

[cit13] Xiang Y., Mei L., Tong A. (2007). Anal. Chim. Acta.

[cit14] Bazzi A., Kreuz B., Wuokila J., Maqboul A. (2005). J. Chem. Educ..

[cit15] Tyagi A. K., Ramkumar J., Jayakumar O. D. (2012). Analyst.

[cit16] Cox J. A., West J. L., Kulesza P. J. (1984). Analyst.

[cit17] Guzinski M., Lisak G., Kupis J., Jasinski A., Bochenska M. (2013). Anal. Chim. Acta.

[cit18] Jasiński A., Guziński M., Lisak G., Bobacka J., Bocheńska M. (2015). Sens. Actuators, B.

[cit19] Lisak G., Sokalski T., Bobacka J., Harju L., Mikhelson K., Lewenstam A. (2011). Anal. Chim. Acta.

[cit20] Fanjul-Bolado P., Hernandez-Santos D., Lamas-Ardisana P. J., Martın- Pernıa A., Costa-Garcıa A. (2008). Electrochim. Acta.

[cit21] Lu Z., Lin X., Zhang J., Dai W., Liu B., Mo G., Ye J., Ye J. (2019). Electrochim. Acta.

[cit22] Liu C., Jiang X., Zhao Y., Jiang W., Zhang Z., Yu L. (2017). Electrochim. Acta.

[cit23] Abbas A. A. (2004). Synthesis.

[cit24] Ali T. A., Mohamed G. G., Farag A. H. (2015). Int. J. Electrochem. Sci..

[cit25] Ali T. A., Mohamed G. G., Said H. A. (2016). Chem. Eng. Commun..

[cit26] Ali T. A., Mohamed G. G., Azzam E. M. S., Abd-Elaal A. A. (2014). Sens. Actuators, B.

[cit27] Kamata S., Bhale A., Fukunaga Y., Murata A. (1998). Anal. Chem..

[cit28] Rebary B., Paul P., Ghosh P. K. (2010). Food Chem..

[cit29] Pechenkina I. A., Mikhelson K. N. (2015). Russ. J. Electrochem..

[cit30] Khani H., Rofouei M. K., Arab P., Gupta V. K., Vafaei Z. (2010). J. Hazard. Mater..

[cit31] Soleymanpour A., Ghasemian M. (2015). Measurement.

[cit32] Ali T. A., Mohamed G. G., Omar M. M., Hanafy N. M. (2017). J. Ind. Eng. Chem..

[cit33] Perez M. A. A., Marin L. P., Quintana J. C., Pedram M. Y. (2003). Sens. Actuators, B.

[cit34] Girault H. H. , Electrochemistry at liquid–liquid interfaces, in Electroanalytical Chemistry, ed. A. J.Bard, C. G.Zoski, Taylor & Francis, Boca Raton, 2010, vol. 23, p. 1

[cit35] Mahajan R. K., Sood P. (2007). Int. J. Electrochem. Sci..

[cit36] Shamsipur M., Kazemi S. Y., Shargi H. (2007). Sensors.

[cit37] Bakker E., Buhlmann P., Pretsch E. (1997). Chem. Rev..

[cit38] Umezawa Y., Umezawa K., Sato H. (1995). Pure Appl. Chem..

[cit39] Gupta V. K., Sethi B., Sharma R. A., Agarwal S., Bharti A. (2013). J. Mol. Liq..

[cit40] Aglan R. F., Mohamed G. G., Mohamed H. A. (2012). J. Pharm. Res..

[cit41] Deshmukh M. A., Celiesiute R., Ramanaviciene A., Shirsat M. D., Ramanavicius A. (2018). Electrochim. Acta.

[cit42] Ivari S. A. R., Darroudi A., Zavar M. H. A., Zohuri G., Ashraf N. (2017). Arabian J. Chem..

[cit43] Rezayi M., Heng L. Y., Kassim A., Ahmadzadeh S., Abdollahi Y., Jahangirian H. (2012). Sensors.

[cit44] Ghaedi M., Naderi S., Montazerozohori M., Taghizadeh F., Asghari A. (2017). Arabian J. Chem..

[cit45] Ardakani M. M., Kashani M. K., Kashani M., Ensafi A. A. (2005). Sens. Actuators, B.

[cit46] IUPAC (1979). Analytical chemistry division, commission on analytical electrodes. Pure Appl. Chem..

[cit47] Frag E. Y. Z., Mohamed M. E., Ali A. E., Mohamed G. G. (2020). Ind. J. Chem. A.

[cit48] Abu Shawish H. M., Elhabiby M., Abu Aziz H. S., Saadeh S. M., Tbaza A. (2016). Sens. Actuators, B.

[cit49] Gupta V. K., Singh L. P., Singh R., Upadhyay N., Kaur S. P., Sethi B. (2012). J. Mol. Liq..

[cit50] Yuan Y. and Lee T. R., Contact Angle and Wetting Properties, in Surface Science Techniques Springer Series, Surface Sciences, ed. G.Bracco and B.Holst, Springer Berlin Heidelberg, Berlin and Heidelberg, 2013, vol. 51, pp. 3–34

[cit51] Huang M. R., Rao X. W., Li X. G., Ding Y. B. (2011). Talanta.

[cit52] Wilson D., Arada M. A., Alegret S., Valle M. (2010). J. Hazard. Mater..

[cit53] Li X. G., Ma X. L., Huang M. R. (2009). Talanta.

[cit54] Abbaspour A., Mirahmadi E., Khalafi-nejad A., Babamohammadi S. (2010). J. Hazard. Mater..

[cit55] Zare H. R., Ardakani M. M., Nasirizadeh N., Safari J. (2005). Bull. Korean Chem. Soc..

[cit56] Yaftian M. R., Rayati S., Emadi D., Matt D. (2006). Anal. Sci..

[cit57] Muller B., Hauser P. C. (1996). Anal. Chim. Acta.

[cit58] Luo X., Huang W., Shi Q., Xu W., Luan Y., Yang Y., Wang H., Yang W. (2017). RSC Adv..

[cit59] Lan T., Furuya K., Lu Y. (2010). Chem. Commun.

